# A new approach to characterize cardiac sodium storage by combining fluorescence photometry and magnetic resonance imaging in small animal research

**DOI:** 10.1038/s41598-024-52377-w

**Published:** 2024-01-29

**Authors:** Martin Christa, Franziska Dithmar, Tobias Weinaus, Michael Kohlhaas, Anahi-Paula Arias-Loza, Michelle Hofmann, Ibrahim A. Elabyad, Fabian T. Gutjahr, Christoph Maack, Wolfgang R. Bauer

**Affiliations:** 1 Comprehensive Heart Failure Center, University and University Hospital Würzburg, Würzburg, Germany; 2 Department of Internal Medicine I, University Hospital Würzburg, Oberdürrbacher Straße 6, Haus A3, 97080 Würzburg, Germany; 3 Experimental Physics 5, University Würzburg, Würzburg, Germany

**Keywords:** Preclinical research, Translational research, Cardiology

## Abstract

Cardiac myocyte sodium (Na^+^) homoeostasis is pivotal in cardiac diseases and heart failure. Intracellular Na^+^ ([Na^+^]_i_) is an important regulator of excitation–contraction coupling and mitochondrial energetics. In addition, extracellular Na^+^ ([Na^+^]_e_) and its water-free storage trigger collagen cross-linking, myocardial stiffening and impaired cardiac function. Therefore, understanding the allocation of tissue Na^+^ to intra- and extracellular compartments is crucial in comprehending the pathophysiological processes in cardiac diseases. We extrapolated [Na^+^]_e_ using a three-compartment model, with tissue Na^+^ concentration (TSC) measured by in vivo ^23^Na-MRI, extracellular volume (ECV) data calculated from T1 maps, and [Na^+^]_i_ measured by in vitro fluorescence microscopy using Na^+^ binding benzofuran isophthalate (SBFI). To investigate dynamic changes in Na^+^ compartments, we induced pressure overload (TAC) or myocardial infarction (MI) via LAD ligation in mice. Compared to SHAM mice, TSC was similar after TAC but increased after MI. Both TAC and MI showed significantly higher [Na^+^]_i_ compared to SHAM (around 130% compared to SHAM). Calculated [Na^+^]_e_ increased after MI, but not after TAC. Increased TSC after TAC was primarily driven by increased [Na^+^]_i_, but the increase after MI by elevations in both [Na^+^]_i_ and [Na^+^]_e_.

## Introduction

Heart failure (HF) with reduced ejection fraction (HFrEF) activates the renin–angiotensin–aldosterone system (RAAS) as a compensatory mechanism. The sustained activation of the RAAS in heart failure can result in increased fluid retention, increased Na^+^ retention and thus, an imbalance Na^+^ homeostasis. Elevated intracellular Na^+^ concentrations ([Na^+^]_i_) in failing cardiac myocytes^1^, seen in pressure overload^2^ or myocardial infarction models^3^, increase cytosolic Ca^2+^ ([Ca^2+^]_c_) but impede mitochondrial Ca^2+^ accumulation, leading to an energetic deficit and oxidative stress^3–5^.

An evolving concept for cardiac diseases and HF regarding extracellular Na^+^ ([Na^+^]_e_) is the (water free) extracellular storage, with Na^+^ ions bound to negatively charged glycosaminoglycans (GAG) in the extracellular space^5,6^. Recently, Artyukov et al.^7^ exploited high-resolution X-ray fluorescence (XRF) spectromicroscopy to visualize this glycosaminoglycan bound to (extracellular) Na^+^ in tissue samples. While it is well established that in heart failure, [Na^+^]_i_ is increased and impacts excitation–contraction coupling and mitochondrial energetics^1^, the role of [Na^+^]_e_ in cardiac dysfunction and failure still needs to be explored in more details.

It is currently hypothesized that the GAG-related storage is supposed to even out elevated (plasma and unbound extracellular) Na^+^ concentrations, preventing water retention and keeping up normal transmembrane gradients between the intra- and extracellular compartments. High concentrations of unbound [Na^+^]_e_ promote GAG synthesis and cross-linking, thus increasing the capacity of the extracellular Na^+^ storage^8^. This increase in extracellular matrix proteins like collagen fibers and GAG is also recognized as diffuse fibrosis—a feature seen in patients with HF with preserved^9^ or reduced EF^10^. It has deleterious mechanical consequences, namely ventricular stiffening and impaired cardiac (diastolic) function (reviewed by Schimmel et al.^11^). Thus, increased diffuse fibrosis observed in the remote myocardium after MI or in pressure overloaded hearts^12^ might also act as additional [Na^+^]_e_ storage, because of the associated increased GAG content.

Currently, various approaches are in use to evaluate Na^+^ levels in an organism. For indirect characterization of the Na^+^ balance in population studies, Na^+^ intake and urinary Na^+^ excretion are used but are merely an approximation^13^ and difficult to implement regarding the monitoring of salt and/or Na^+^ intake. When working with tissue samples or organoids, flame atomic absorption spectrometry is an accurate method to detect total tissue Na^+^ content, but not intra- or extracellular fractions. This is done at the expense of the sample^14,15^, thus not used in clinical practice but in analyses for industry or food chemistry. In preclinical research, fluorescence dye-based photometry is a widespread standard method to quantify [Na^+^]_i_ in isolated cells. In most cases, sodium binding benzofuran isophthalate (SBFI) is used as a ratiometric Na^+^ sensitive fluorescent dye with a decent selectivity for Na^+^ in the presence of other physiological ions^16,17^.

In recent years, Na^+^ MRI has gained attention in small animal research and medicine. Latest hardware developments, like higher field strength available, improved (cryo-)coils, and dedicated sequences allow non-invasive detection of tissue sodium concentration (TSC) in reasonable scan-time. Various tissues, including the brain^18^, skeletal muscle^19,20^, skin^19,21–23^, tumors^24^, cartilage^25^ and heart^19,26^ were investigated for TSC changes. Quantifying Na^+^ via MR requires ultra-short echo time (UTE) sequences due to the rapid T2 signal decay (i.e. the signal is disappearing fast) and benefits from higher field strength (3 Tesla, preferably 7 Tesla). However, low signal-to-noise ratio and uncertain relaxation properties of Na^+^ compartments pose challenges and make it highly difficult to attribute fractions of the signal either to the intra- or extracellular space^27^. Techniques like inversion pulses^28^ and quantum filtering^29,30^ have been proposed for [Na^+^]_i_ quantification, and validated with sophisticated experiments, e.g., in Langendorff-perfused hearts^2,31^. Other studies showed a relevant impact of the extracellular compartment on the signal of double or triple quantum filter, thus signal allocation with these techniques remains under debate^27,32,33^. Additionally, these refinements decrease signal intensity^34^ and increase measurement time. This leaves UTE-based TSC quantification via ^23^Na MRI as the most feasible in vivo approach for living mice available today.

**Figure 1:**
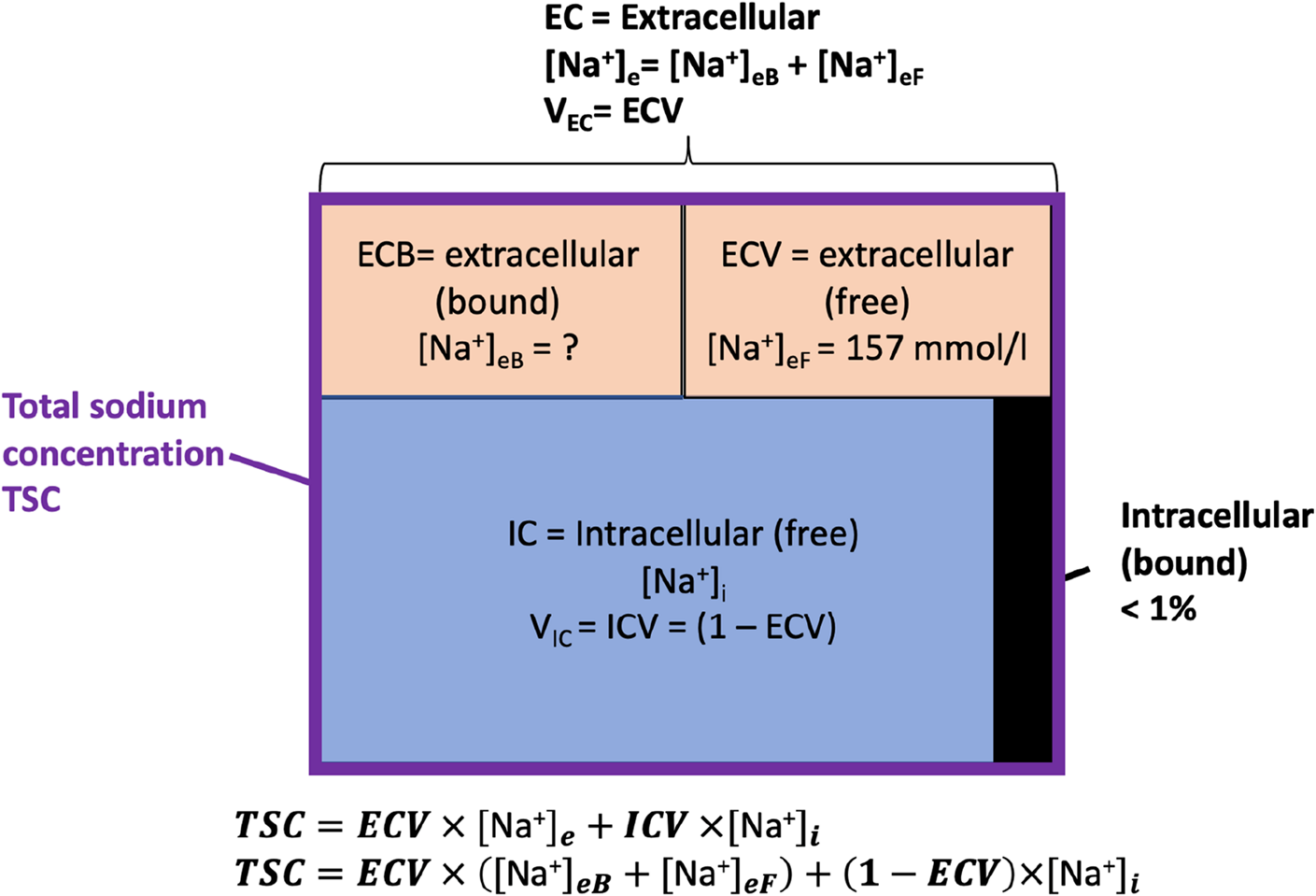
Compartment model of an imaging voxel. Each voxel in the cardiac tissue is constituted by intra- and extracellular space in different proportions. Each compartment contains free and bound sodium ions. One can assume a fixed concentration of extracellular free sodium ions (157 mmol/l), as this compartment is in equilibrium with the blood serum sodium. TSC is quantified in vivo by ^23^Na-MRI measurements and after sacrificing the animals, intracellular sodium concentration is measured by fluorescence microscopy. The fraction of intracellular bound sodium is below 1% and thus neglected. Other models additionally consider a ”fat compartment”, which is relevant in e.g. breast tissue or skin, but not in the myocardium. This simplifies calculation of [Na^+^]_e_. *TSC* total sodium concentration (per Voxel), *IC* Intracellular, *ICV* Intracellular Volume, *EC* extracellular, *ECV* extracellular Volume.

To have a tool to tackle the above-mentioned questions on intra- and extracellular Na^+^ distribution and disease-related changes in the heart, we decided to combine UTE-based TSC quantification and extracellular volume measurements via MR imaging, and intracellular Na^+^ levels via fluorescence microscopy—a method widely accepted in the field of cellular research to quantify [Na^+^]_i_^3,17,35^. For in vivo MRI measurements, the Na^+^ signal of the tissue is calibrated to the signal of the reference tubes to allow quantification. In fluorescence microscopy measurements, a calibration curve is generated for each run to calculate Na^+^ based on the measured fluorescence signal of the cardiomyocytes. Based on a multi-compartment model (as illustrated in Fig. 1), incorporating these parameters (TSC, ECV and [Na^+^]_i_) the obtained data allows deriving extracellular Na^+^ levels.

Theoretically, one voxel can consist of three compartments (compare Fig. 1), contributing to the total sodium signal. These compartments are (1) intracellular, (2) extracellular and (3) fat, whereas (1) and (2) may be further differentiated into a (a) bound and (b) free Na^+^ compartment. While (3) fat in the heart is mainly found in the epicardial and perivascular compartments^36^, these do not significantly contribute to the Na^+^ MR signal in the myocardium, but indeed may play a relevant role when investigating other organs.

Concerning the bound and “free” fractions of (1) and (2), Burstein and Springer^27^ revealed that truly bound intracellular Na^+^ (1a) is a negligible part of the Na^+^ signal, where the maximum of “bound” intracellular Na^+^ is around 10^–2^ fmol, representing a fraction of less than 1%. For the extracellular space, levels of Na^+^ are higher, since the free Na^+^ fraction (2b) is in constant exchange with the blood and therefore, in mice equals the blood serum concentration of around 157 mmol/l. The bound Na^+^ fraction (2a) on the other hand can range from zero to a previously unknown concentration, depending on GAG density and crosslinking.

Therefore, according to our model (Fig. 1), any signal value of the extracellular compartment that exceeds normal serum Na^+^ levels of mice (around 157 mmol/l) is attributed to water-free, bound Na^+^ storage in the EC. A very similar model, in which [Na^+^]_e_ was fixed at 140 mmol/l, has already been successfully used by Madelin et al. to calculate [Na^+^]_i_ in breast cancer tissue^24^.

After establishing the workflow, we tested the setup to evaluate the Na^+^ distribution in two different mouse models of cardiac disease: trans-aortic constriction surgery as model for pressure overload and cardiac hypertrophy, and myocardial infarction by LAD ligation as a heart failure model due to a loss of working myocardium and geometric changes of the ventricle.

## Results

### LV remodeling characterized by MRI and histology

**Figure 2:**
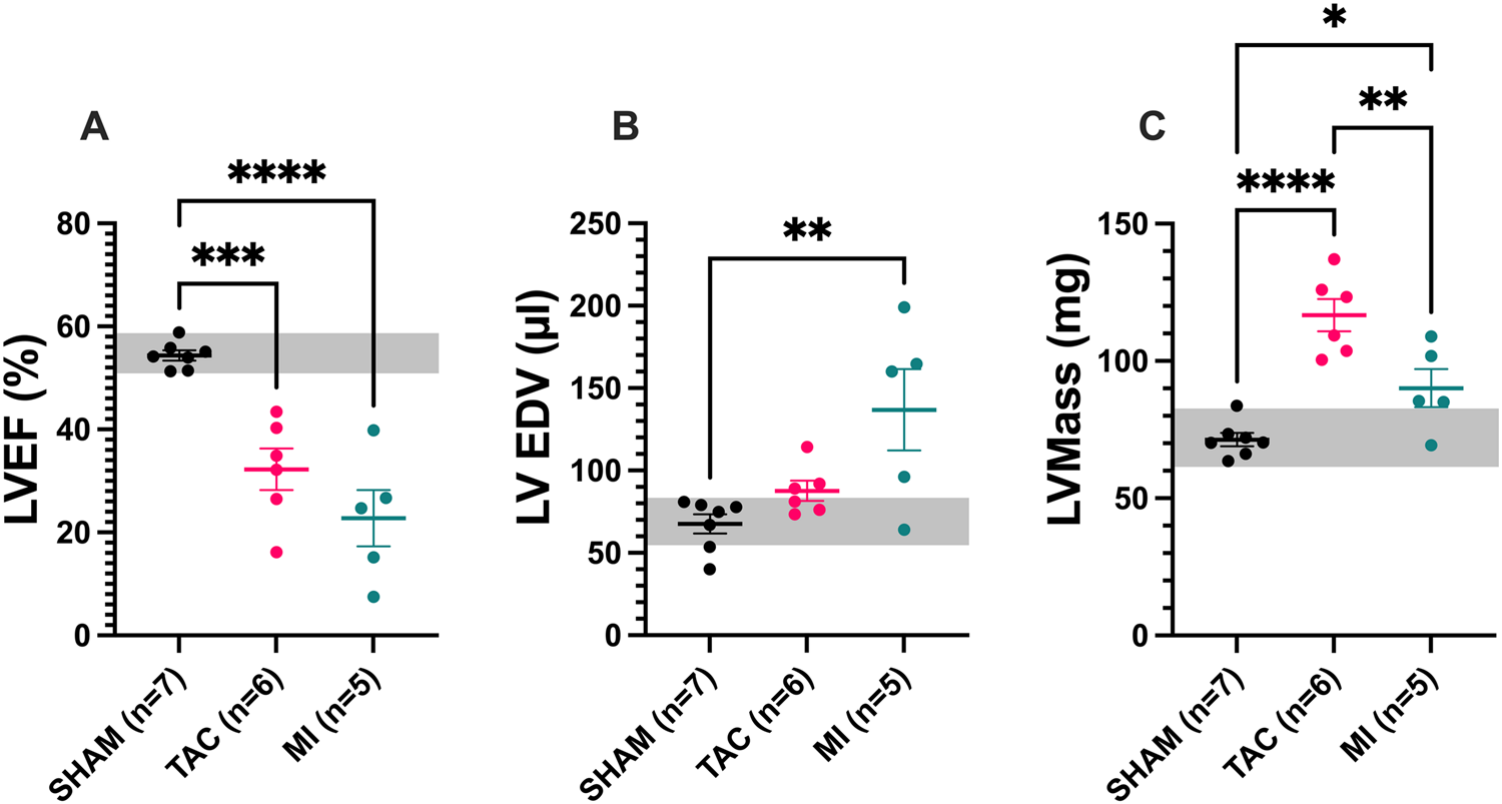
Cardiac functional parameters measured by MRI. MR measurements of left ventricular ejection fraction (LVEF), left ventricular end diastolic volume (LVEDV), and left ventricular mass (LVMass) are presented. TAC and MI animals showed reduced LVEF (**A**). LVEDV was largest in the MI group (**B**), whereas LVmass was highest in the TAC group. (**C**) The grey area represents the 95% confidence interval of values from the SHAM group. *p < 0.05; **p < 0.01; ***p < 0.001; ****p < 0.0001.

Eight weeks after sham, TAC or MI surgery, MRI was performed. Compared to the SHAM group (54 ± 3%), LV ejection fraction decreased in mice after TAC (32 ± 10%) and to a higher extent, also after MI (23 ± 12%; Fig. 2A). Furthermore, LV end-diastolic volume (EDV) increased after MI (137 ± 55 ml), but not after TAC (88 ± 15 ml vs SHAM 68 ± 15 ml; Fig. 2B). In contrast, LV mass increased to a greater extent after TAC (117 ± 14 mg) than after MI (90 ± 16 mg vs SHAM 71 ± 6 mg; Fig. 2C).

Histological analyses of WGA-stained short axes slices revealed that myocyte cross sectional area substantially increased after both TAC and MI, but to a higher degree after TAC compared to MI (Supplement Fig. S1A,B). In contrast, fibrosis (determined by PSR Staining) increased substantially after MI, but not after TAC (Supplement Fig. S1C,D).

### Changes in tissue characteristics (T1 and ECV)

**Figure 3:**
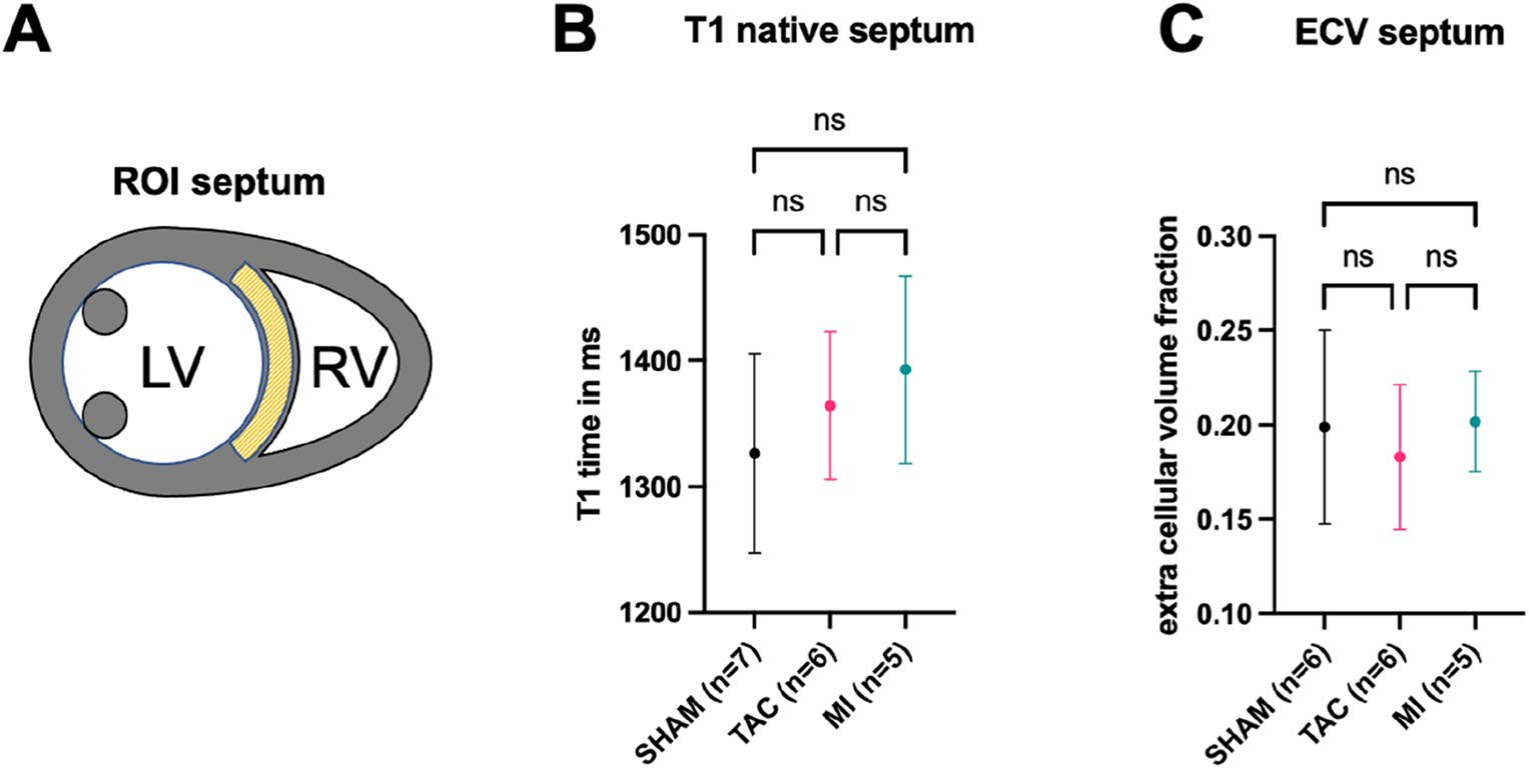
T1 and ECV values. (**A**) is a representation of typical ROI placement in the septum for MRI data analyses. The native T1 values determined in the respective ROI for the individual groups are shown in (**B**). The corresponding ECV results can be found in (**C**). Of note, ECV data is only available from 6 SHAM mice, as contrast agent injection failed in one mouse. ns: p = not statistically significant.

T1 mapping of the myocardium was conducted once prior (native T1) and two times (15 and 25 min) after contrast agent application (post T1) in a mid-ventricular short axis slice. The three maps were used to calculate the extracellular volume (ECV) per voxel. Figure 3A shows representative drawings of the regions of interest (ROI) in the septum, which was chosen to evaluate the tissue characteristics and to quantify total tissue Na^+^ content. Native T1 mapping revealed a non-significant trend towards increased mean T1 values in TAC (1364 ± 59 ms) and MI (1393 ± 74 ms) compared to Sham mice (1327 ± 79 ms), respectively (Fig. 3B). The overall values for ECV in the septum were not significantly different between midventricular SAX slices in Sham, TAC and MI hearts (Fig. 3C).

### Na^+^ measurements via MRI and fluorescence microscopy

**Figure 4:**
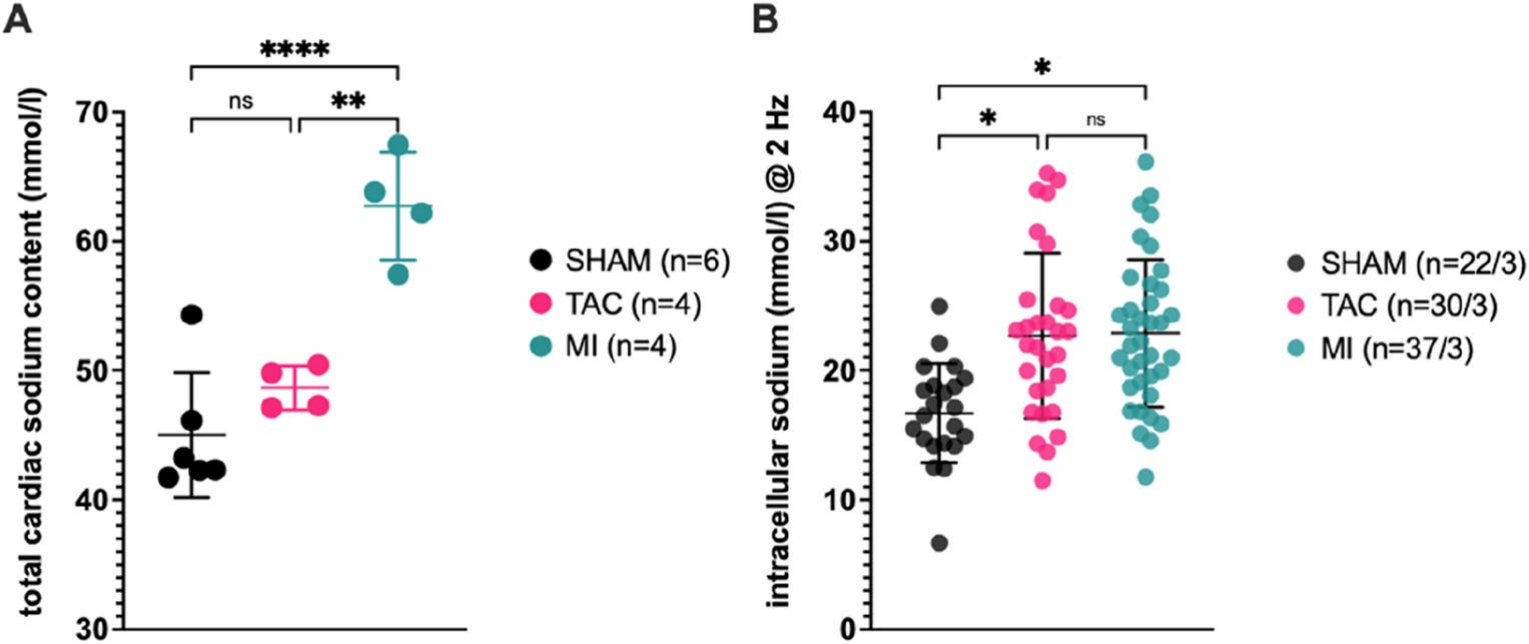
Total myocardial sodium content and intracellular sodium levels. (**A**) Total tissue sodium levels are determined by sodium MRI in the myocardial septum, where the TSC in MI is significantly higher compared to TAC and SHAM. (**B**) Intracellular sodium levels measured via SBFI fluorescence was elevated in TAC and MI compared to SHAM, when stimulated with 2 Hz. For (**B**) significance is reported as results from a nested one-way ANOVA. *p < 0.05; **p < 0.01; ****p < 0.0001; ns: p not statistically significant.

**Figure 5:**
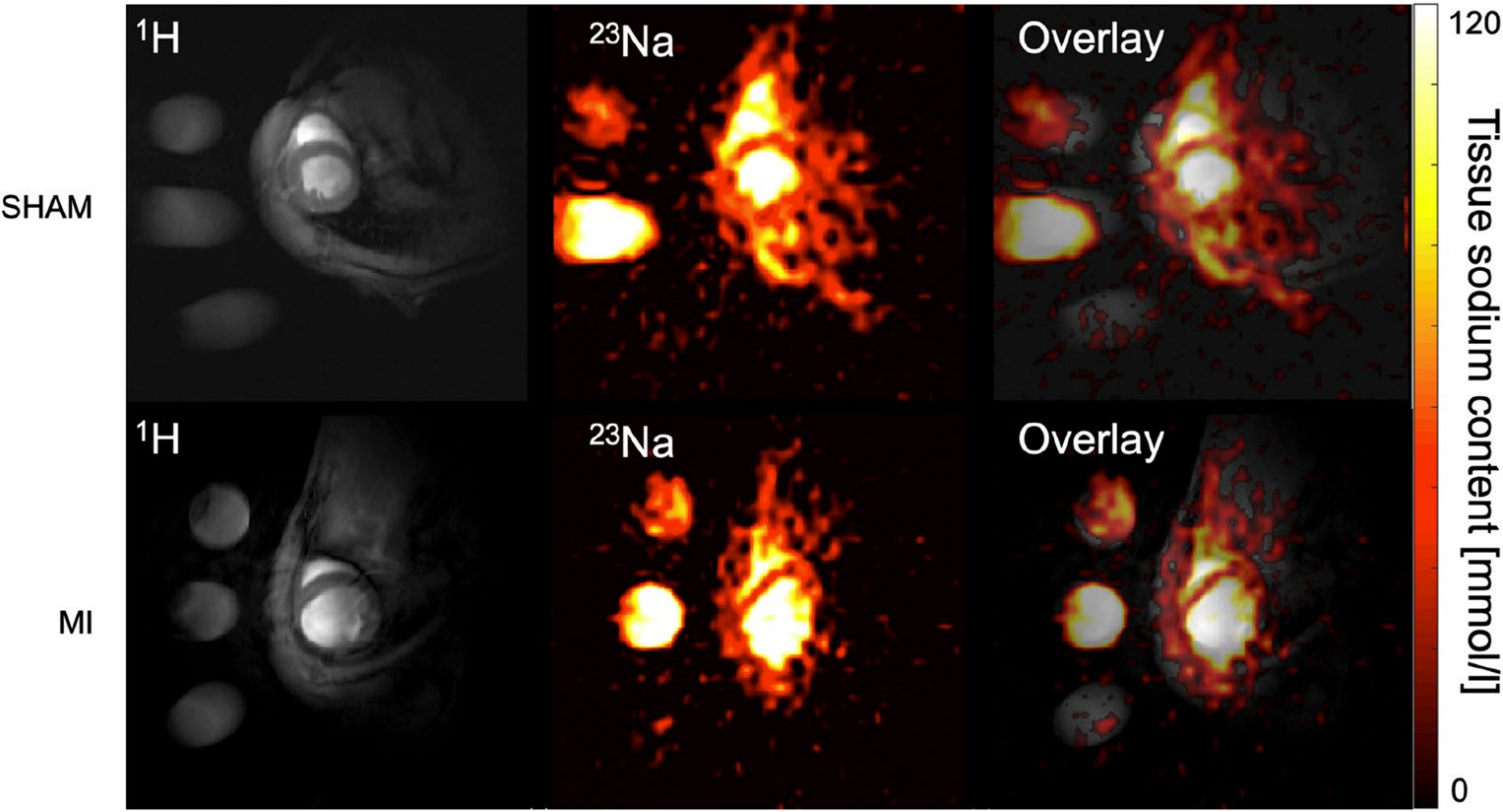
Comparing SHAM and MI proton and sodium short axis images. This panel shows the respective midventricular short axis slice acquired as proton image (left column) and the corresponding sodium image (middle column) as well as the fusion of both (right column). In the proton MI SAX, the thinned myocardium and the dilated left ventricle. In the sodium images the two reference vials (100 mmol/l—the tube with the higher signal, and 50 mmol/l—the round slightly weaker signal above the 100 mmol/l tube) are clearly distinguishable.

Measurements of total tissue Na^+^ concentrations (TSC) were conducted in the myocardial septum to reduce motion artifacts, and the regions of interest were automatically trimmed by a custom algorithm prior to analysis to avoid partial volume effects at the myocardium-blood border. The ROI placement in the septum was the same as for T1 and ECV quantification (Fig. 3A). Compared to sham (45.0 ± 4.8 mmol/l), TSC substantially increased after MI (62.7 ± 4.2 mmol/l; p < 0.0001), but only modestly after TAC (48.7 ± 1.7 mmol/l; p = n.s.; Fig. 4A). A representative comparison of SHAM and MI Na^+^ images is shown in Fig. 5.

In cardiac myocytes isolated from the hearts of mice, the intracellular Na^+^ concentration ([Na^+^]_i_) at a stimulation rate of 2 Hz was higher after both TAC (22.7 ± 6.4 mmol/l; p = 0.006) and MI (22.9 ± 5.7 mmol/l; p = 0.0008) compared to SHAM (16.7 ± 3.8 mmol/l), with no differences between TAC and MI myocytes, respectively (Fig. 4B).

### Comparing [Na^+^]_i_ against TSC

**Figure 6:**
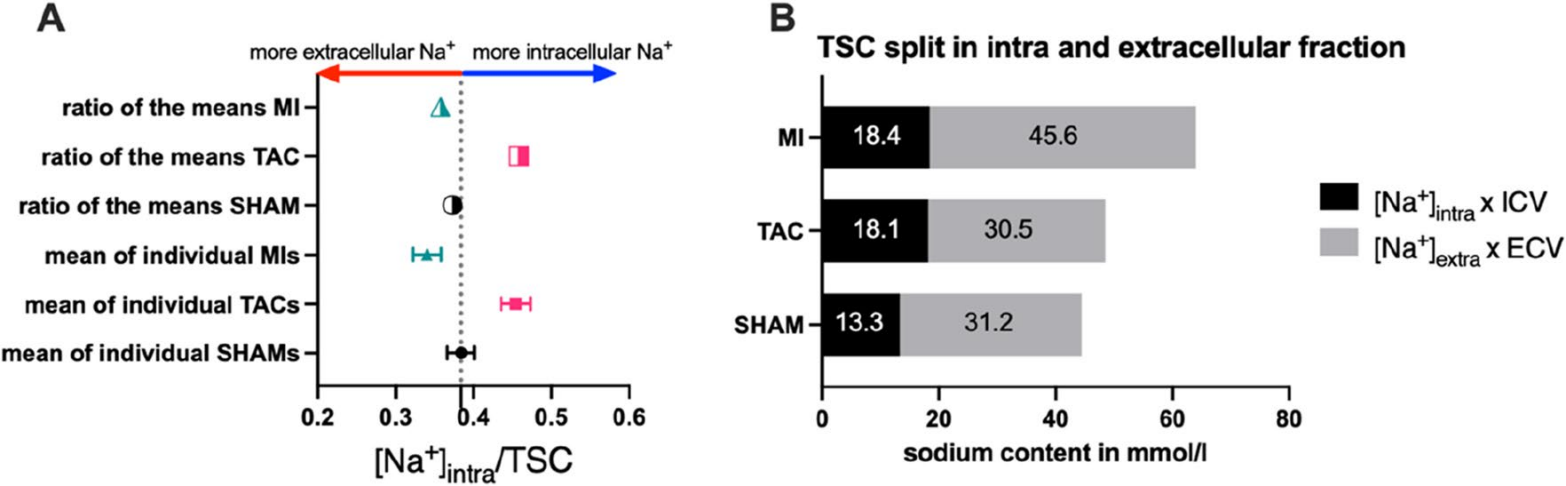
Comparison of TSC against intracellular sodium levels and representation of the calculated intra- and extracellular fraction of the TSC signal. (**A**) shows the ratio [Na^+^]_intra_/TSC, with a higher value representing a greater dependence of TSC on intracellular sodium. A lower ratio shows a greater dependence on extracellular sodium content. (**B**) shows the calculated contribution of the intra- or extracellular sodium content (in mmol/l) to the total TSC signal per voxel.

To characterize the relationship between TSC and [Na^+^]_i_, we calculated the ratio between the two parameters based on the mean values presented above. For the calculation incorporating all data, the [Na^+^]_i_/TSC ratio of the *mean* values in SHAM is 0.373, for TAC 0.458 and for MI 0.358. Looking at individual mice, from which both TSC and [Na^+^]_i_ were available (n = 3 per group), the mean of the individual ratios for SHAM mice were 0.384, for TAC 0.454 and for MI 0.345 (Fig. 6A). The single values for each mouse are listed in the supplement (Table ST1A–C).

### Calculated extracellular Na^+^ levels

Using [Na^+^]_i_ and proton-MRI derived ECV-values, we calculated extracellular [Na^+^] concentrations ([Na^+^]_e_) for each group based on the mean values, and again for each individual mouse if the necessary variables were available. [Na^+^]_e_ calculated from mean values out of all available data was 157.1 mmol/l after Sham, 163.4 mmol/l after TAC and 230.3 mmol/l after MI.

Additionally, we used this data to determine the contribution of the intra- and extracellular compartments to the TSC. Therefore, we calculated the intracellular Na^+^ fraction ([Na^+^]_i_ × (1 − ECV)) and the extracellular Na^+^ fraction ([Na^+^]_e_ × ECV). The extracellular component accounted for 70% of the TSC signal in the SHAM group, 63% in the TAC group and 71% in the MI group. A visualization of the proportion of the respective component (intracellular or extracellular) in the mean total signal/sodium content of a voxel can be found for each group in Fig. 6B.

## Discussion

The current study combines the advantages of two techniques, namely UTE based in vivo quantification of total tissue Na^+^ levels via MRI and determination of intracellular Na^+^ concentrations in isolated cardiomyocytes via fluorescence dye-based photometry, to characterize tissue Na^+^ storage in different mouse models of cardiovascular disease. By employing MRI derived information on the extent of the extracellular volume, we calculated extra-cellular Na^+^ levels, in addition to the directly measured intracellular and total Na^+^ levels, according to our compartment model (Fig. 1). We used this information to characterize the Na^+^ storage in mice that underwent either SHAM, TAC or MI surgery and showed an increased TSC in the remote myocardium of MI mice, due to an increased *extracellular* Na^+^ storage and also *increased* intracellular Na^+^ levels. In contrast, the slightly elevated TSC in TAC mice was mainly explained by higher *intracellular* Na^+^ levels but merely unchanged *extracellular* Na^+^ storage. Thus, our findings suggest that intracellular and extracellular Na^+^ distribution and their disease-related changes differ with respect to the (experimenta)l cause of cardiac damage.

Single cell experiments reported increased intracellular Na^+^ ([Na^+^]_i_) in cardiac myocytes from patients with HFrEF^3^. Pieske et al. showed an increase in [Na^+^]_i_ accompanied by a decline in isometric force in failing human cardiomyocytes^37^. These observations were confirmed recently by Akentijevic et al., who showed an approx. 40% increase of [Na^+^]_i_ and a decline in in vivo function in a mouse model of cardiac hypertrophy^2^. Our reported results for [Na^+^]_i_ levels (e.g. Sham 16.7 ± 3.8 mmol/l vs 22.9 ± 5.7 mmol/l in MI cardiomyocytes) are in line with theses previous publications with mean [Na^+^]_i_ levels increasing by 37% (mean MI [Na^+^]_i_ compared to mean Sham [Na^+^]_i_) and 36% (TAC vs Sham) in isolated cardiomyocytes, respectively. However, changes in septal myocardial TSC were + 39% in MI (62.7 ± 4.1 mmol/l against 45.0 ± 4.8 mmol/l in SHAM), but only + 8% in TAC compared to SHAM animals. Additionally, our mouse models showed the expected decline in in vivo myocardial function, as represented by reduced left ventricular ejection fraction (LVEF). This is also in agreement with the mentioned studies, as they correspondingly showed metabolic alterations triggered by chronic elevated [Na^+^]_i_, leading to an energetic impairment of cardiomyocytes and a decline of in vivo function^2^.

External water free storage, demonstrated by Machnik et al.^8^ and Titze al.^38^ is today a commonly accepted explanation how various tissues store Na^+^ in an osmotically inactive form. Many cardiac pathologies are accompanied by diffuse fibrosis from early stages on. According to Nijst et al.^6^, extracellular Na^+^ itself is a trigger for increased poly-glycosamino-glycane (GAG)-crosslinking, thus a positive feedback loop to increase storage capacity. In the normal state, high Na^+^ concentration leads to changes in the GAG sulfation pattern and increases GAG charge density^6^. Due to increased GAG synthesis and cross linking, the EC space becomes more “tightly” packed, but does not increase its volume—the compensated state (Fig. 1; [Na]_e_ increases due to an increase in [Na]_e_bound_ and constant [Na]_e_free_). In the decompensated state, the GAG network is not able to balance out the changes in Na^+^, therefore water (and increasing oncotic pressure) is following the free Na^+^ ions, leading to an increase in ECV—[Na]_e_ rises further as [Na]_e_bound_ has reached its upper capacity limit, and [Na]_e_free_ is rising beyond 157 mmol/l.

The increase in GAG molecules and crosslinking of GAG additionally leads to myocardial stiffening. In our case, TAC animals had near to normal extracellular Na^+^ levels and showed an impairment of LVEF. The decline in LVEF may be a long-term consequence of cardiac remodeling, which typically starts with myocyte hypertrophy, as seen in our histological analyses, and which is commonly associated with an energetic deficit, which to some extent is related to increased intracellular Na^+^ levels^2,37^—representing the state of decompensated cardiac hypertrophy. In contrast, in MI animals—showing [Na^+^]_i_ comparable to levels in TAC cardiomyocytes—the reduced LV function in MI animals may not exclusively be attributed to the infarction scar (replacement fibrosis) and macro-geometric changes in the ventricle, but also to a stiffening in the remote (thus presumably healthy) myocardium and altered mechanical characteristics due to the increased [Na^+^]_e_ and GAG crosslinking, and thus due to diffuse fibrosis. The tendency to higher T1 times (often seen as surrogate for diffuse fibrosis) in the septal remote myocardium of MI animals compared to the other groups supports this hypothesis.

Commonly the TAC model is known for myocyte hypertrophy and accompanied fibrosis. Our ECV data of the septum correlated well with position matched histological findings, also showing no difference between TAC and SHAM. Keeping in mind that ECV is a volume fraction, it can also be changed indirectly by changes in size of the intracellular volume. With the strong myocyte hypertrophy in TAC (compare myocyte cross sectional areas in Fig. S1A,B), the significant increase in ICV could be the explanation for the “relatively” unchanged or slightly lower septal ECV (Fig. 3C) in relation to the total voxel volume. This effect of indirect ECV change can be observed, for example, in the athlete’s heart, where myocyte hypertrophy leads to a decrease in ECV^39^.

Another explanation might be the choice of needle diameter in TAC surgery, as it can affect the outcome. In our TAC model and around 75% of the reported cases, a 27G needle is used, but technical issues during surgery can lead to a larger final diameter (comparable to 26G or 25G)^40^. Richards et al.^41^ evaluated three TAC phenotypes based on needle diameter (25G, 26G, or 27G). Only the 26G and 27G groups showed increased myocyte size, while interstitial fibrosis was only significant in the 27G group. MRI measurements were not performed in Richards' study, so direct ECV value comparison is not possible. Coelho-Filho et al.^42^ reported an ECV of 25% in healthy animals, and showed that an increase in cellular volume precedes fibrosis in TAC mice. Thus, TAC mice in our study either may have insufficient narrowing (closer to 26G than 27G), explaining the low amounts of fibrosis (Fig. 2C,D) or had only developed myocyte hypertrophy but not yet fibrosis. Other studies reported ECV values for healthy mice of 22 ± 2%^43^, which is in concordance with our mean ECV in healthy mice of around 20%. Also an increased myocardial mass, an impairment of cardiac function and a slight increase in native T1 values similar to the observed changes in our experiments (Fig. 3) was shown^12^.

Of note, mid-ventricular short axes slices were chosen for T1 mapping and ECV calculations and the region of interest was deliberately placed in the remote myocardium—the septum, to analyze these parameters at the same location where TSC was quantified. Thus, MI animals did also not show significantly higher ECV values in the septum. Of note, the histological analyses of PSR stained mid ventricular sections (Fig. S1C) did not show a significant increase in fibrosis in the total myocardium of TAC animals, but in MI mice (Fig. S1D), as the entire cross-section was analyzed and not just the ROI in the septum as chosen for ECV.

### Limitations

Our experimental setup used a custom-made saddle-shaped surface coil to (a) optimally cover the thorax of a mouse, (b) to provide good penetration and SNR for Na^+^ measurements and (c) allow acquisition of proton and Na^+^ images without changing the coil or repositioning of the animal. Thus, the vendors adiabatic hyperbolic secant pulse was used for inversion. Adiabatic pulses are less sensitive to B1-inhomogeneity, ensuring a full inversion over the region of interest for T1 mapping. Quality of the acquired ECV maps is thus depending on the coil’s penetration of the respective mouse and the quality of the adiabatic pulses. Regarding sodium MRI, due to B1 field inhomogeneities (which have been corrected for), quantum filter preparations are not possible with the surface coil. Furthermore, due to the low SNR of ^23^Na and the resulting low resolution, Na^+^ levels may be overestimated in MI animals, as with thinned myocardial walls and a constant voxel size, the partial-volume (PV) effect supposedly has a stronger influence. Lott et al. suggested a sophisticated PV correction algorithm for images acquired with volume coils^44^, whereas we used a dedicated inhouse developed script, to reduce the manually drawn ROIs to ensure only signal from the myocardium is analyzed. The sodium signal needs to be corrected for respiratory and cardiac motion; thus, we used a prospective gating and applied B1 field correction prior calibration and TSC quantification. However, some overestimation of the sodium signal is to be expected if incorrected gating occurs^45^.

When combining different methods of measurement, error propagation needs to be considered. For fluorescence-based Na^+^ measurements the SBFI ratio is theoretically not influenced by the size of the cell and the respective loading and thus only dependent on the actual free ion concentrations (of Na^+^). A fixed concentration is given by the 0 mM Na^+^ calibration solution. We calculated the error (as SD/mean × 100) for the cells (n = 89) of the animals presented in this paper, which results in and potential error of 5.5 ± 2.0%. ECV calculation is based on native T1 and the change of this T1 value after administration of contrast agent. In human MRI, ECV calculations are corrected by hematocrit (Hkt), a blood marker that was not available for our mice. Thus, we fixed this value to 0.55 according to literature. In a similar manner, as described above we calculated that different Hkt values introduce an error of max 18% for ECV, but only 4% for ICV (1 − ECV). The error in TSC detection is represented by the mean noise levels over all groups/sodium measurements. Noise is defined as standard deviation of the background signal. With this, error of TSC detection is around 9%. Therefore, error propagation leads to an uncertainty of 27% in [Na^+^]_e_ calculations. This underlines the need to minimize the error in the TSC determination for future studies and to eliminate the uncertainty in the ECV calculations by direct determination of the blood hematocrit. (Error propagation is shown in the Supplement Table ST2).

Finally, only three animals per group had all necessary measurements available to calculate individual [Na^+^]_e_. Although power calculations resulted in a power of 0.99785, the results of this pilot study primarily underline the applicability of our proposed approach. However, larger animal cohorts will be required to obtain more robust conclusions in future studies.

### Perspective

Measurements of TSC and functional parameters of cardiac MRI were combined with intracellular Na^+^ measurements on isolated CM. These measurements on isolated CM additionally allow to determine parameters like single cell calcium levels, sarcomere length and fractional shortening, as well as the levels of energy equivalents and the cell’s redox state. While triple or double quantum-filters are subject to ongoing discussion and require sophisticated pulse sequences and near to perfect experimental setups, UTE based TSC quantification can be performed with most of the available small animal MR scanners within a reasonable scan time. Newer MRI sequences like T1rho-mapping—which demonstrated to be sensitive for larger macromolecules like GAG or collagen^46^—will improve myocardial tissue characterization, regarding the molecules influenced by Na^+^.

Combining the acquired MR information with the data derived from the comprehensive single cell analyses with broadly available modern fluorescence microscopy grants a wide-ranging characterization of the effects of an intervention or pathological model and thus enables a thorough understanding of the underlying processes. Our setup allows future experiments to investigate the effects of different drugs interfering with Na^+^ homeostasis and deposition, such as SGLT2-inhibitors, spironolactone and NHE inhibitors and to compare their effects on the cardiac Na^+^ storage in health and disease.

## Materials and methods

### Animal experiments

All animal procedures were approved by an institutional review board (District Government of Lower Franconia—Approval RUF55.2.2-253-2-735) and conducted in accordance with institutional guidelines and in compliance with ARRIVE guidelines. C57BL/6N mice were obtained from Charles River (BL/6N, C57BL/6NCrl, strain code 027) and were housed under standard conditions with free access to food and tap water.

### Transaortic constriction and myocardial infarction surgery

A total of 34 male B6/N mice underwent surgery (10 TAC, 16 MI, 8 SHAM). Mice (10 weeks, BW: 22–24g) were anesthetized with buprenorphine (0.1 mg/kg i.p.) and an isoflurane-based inhalation anesthesia (Isofluran evaporator, with 1.5–2% Isoflurane and an oxygen flow rate of 0.5 l/min). After orotracheal intubation using a 20 gauge catheter, the tube was connected to a volume cycled rodent ventilator (Harvard Apparatus, Holliston, MA) on supplemental oxygen with a tidal volume of 0.2 ml and respiratory rate of 120 strokes/min. For trans-aortic constriction (TAC), the chest cavity was accessed via the second intercostal space at the left upper sternal border through a small incision and aortic constriction was performed by tying a 7–0 nylon suture ligature against a 27 gauge needle to yield a narrowing in diameter as a transverse aortic constriction of 65–70%. Control mice underwent a sham operation in which the nylon suture was placed, but not tied.

For MI induction, we performed a left sided thoracotomy between the 3rd and the 4th rib. After opening the pericardium, a surgical ligation was placed on the left coronary artery using a 6–0 silk suture, as described by Kolk et al.^47^ with some alterations. In accordance with the 3R principles, SHAM mice from the TAC and MI groups were pooled. After 8 weeks number of animals per group were SHAM: n = 7; TAC: n = 6; MI: n = 5, (one additional MI mouse was analyzed in histology but did not undergo MRI). A flow chart showing the subgroup allocation of the mice, exclusions and resulting different n-numbers per subgroup can be found in the Supplement (Fig. S2).

### Magnetic resonance imaging

Functional cardiac MRI, sodium MRI and T1 mapping was performed on a 7 Tesla small animal MRI system (Bruker BioSpin, Ettlingen), using customized-built dedicated ^1^H/^23^Na surface coil, with a saddle-shape to fit the body of the mouse. The respective channel of the coil (proton or sodium) could be controlled by changing the connection cables, allowing to acquire ^1^H and ^23^Na images within one session, without manipulating the animal. Eight weeks after surgery, we anesthetized the mice with an isoflurane inhalation anesthesia (Induction: 4–5% Isoflurane and maintenance of anesthesia with 1.5–2% Isoflurane and an oxygen flow rate of 1.5 l/min). Eye ointment was applied to prevent drying out. Body temperature was monitored over the measurement and kept constant (36–37 °C) using a heating pad. Heart and breathing rate were monitored throughout the experiment using ECG and a pressure sensor. (All monitoring equipment from SA Instruments, Stony Brook, NY, USA).

### Sodium MRI

Using a standard 4-chamber and 2-chamber view, a midventricular short-axis imaging slice was selected for sodium measurements. The vendors UTE sequence was modified into a 2D radial UTE with the following sequence parameters: TE: 0.345 ms; TR 100 ms, T_readout_: 0.365 ms, AQ bandwidth 65 kHz, 150 projections, FA 90°, FOV 35 × 35 mm^2^, Slice thickness 4 mm, Acquisition-Matrix 48 × 48 resulting in effective resolution of 0.625 × 0.625mm/Voxel; with cardiac and respiratory motion gating acquisition time was around 45 min, dependent on heart/respiratory rate.)

Additionally, the sequence was repeated with FA 45° and half the averages, to acquire data for B1 field correction via the DAM method. Two vials with a sodium concentration of 50 and 100 mmol/l were attached to the coil as reference for total sodium determination.

Using the DAM Method a B1 map was generated and the signal was corrected accordingly^48^. To quantify the sodium content, the image was standardized normalizing the image on the 100 mmol NaCl vial signal only (using the average noise as 0) Regions of interest (ROIs) were placed in the septum as indicated in Fig. 4A. The septal ROI was placed in the center of the septum and algorithmically shrunken by 5% to avoid contamination from blood signal. The advantage using the septum, is the minimization of motion of the septal wall if the cardiac short axis is correctly planned. This additionally reduces motion and partial volume artifacts and thus leads to more accurate results.

### T1 mapping and ECV calculation

T1 mapping with different gadolinium (Gd) contrast concentrations allows ECV quantification. For this T1 Data were acquired using a retrospectively triggered Inversion Recovery Snapshot FLASH (IRSF) before (one map) and after the i.p. administration of Gd (two maps) (Gd agent = Gadovist, Bayer Vital GmbH, Germany). Gd dosage was adjusted to the respective body weight of the mice (final Gd concentration 0.05 mmol/ml; max volume 150 μl). The data was reconstructed using a model-based approach described in Gutjahr et al.^49^.

The reciprocal of T_1_ ${R}_{1}={T}_{1}^{-1}$ is considered to directly correlated to the concentration of the contrast agent. When R_1,tissue_ is plotted over R_1,blood_ a linear relationship exists in which the slope corresponds to the ECV^43^. R_1,tissue_ is extracted from a ROI in the myocardial septum. R_1,blood_ is extracted from a ROI in the left ventricle. To correct for the hematocrit content in the ventricular blood, the resulting slope must be multiplied by (1 − hematocrit in percent/100). A fixed value of Hct = 55% was chosen, and the slope fitted using a linear fit over the three datapoints.

$$ECV=\left(1-Hct/100\right)*\frac{\Delta {R}_{1,tissue}}{\Delta {R}_{1,blood}}$$

The delay (12–15 min) between contrast agent injection and acquisition of the first post contrast T1 map, was used to obtain a multi-slice short axis cine stack of the heart (7 up to 9 slices depending on the size of the heart) for cardiac functional parameters. We used a vendor provided CINE FLASH sequence with a TR: 10 ms (leading to 12–16 frames, depending on the heart rate), Te 1.4 ms; Field of View 35 × 35 mm^2^, acquisition matrix 192 × 192 leading to resolution of 0.182 × 0.182 mm^2^ with a slice thickness of 1 mm; 3 averages; acquisition time for the stack around 12 min.

To analyze functional data (e.g. left and right ventricular function and volumes, as well as cardiac mass) we transferred DICOM images of the SAX stack to a commercially available software (Medis Suite 4.0, Medis medical imaging, Leiden, Netherlands).

LV endo- and epicardial borders were measured in the end-diastolic and end-systolic frame. Two-dimensional areas were multiplied by the interslice distance (1 mm) to compute a volume. Total volumes were calculated by slice summation. Left ventricular ejection fraction (LVEF) is the ratio of as Stroke-Volume (SV) to LVEDV in %, with SV = LVEDV − LVESV. The myocardial mass (LVM) was defined in the end-diastolic frame by subtracting LVEDV from the epicardial volume and multiplying the result by 1.05 g/ml (estimated density of the myocardium).

### Isolation of cardiomyocytes and fluorescence photometry

One day after successful MRI measurements, animals were sacrificed to isolate cardiac myocytes by enzymatic digestion. The protocol for cardiomyocyte isolation is published und described in detail in Nickel et al. and the associated Supplemental Material^50–52^. For single cell experiments an automated fluorescence microscope (IonOptix/CytoCyfer Multicell HTS) was used to measure up to 20 cells per run. For fluorescence based measurements of [Na^+^]_intra_ ventricular myocytes were incubated with Sodium binding benzofuran isophthalate (SBFI) for 60 min at 25 °C. After the transfer into the IonOptix chamber, the chamber was perfused with NT solution (containing [in mM]: NaCl 130, KCl 5, MgCl2 1, CaCl_2_ 1, Na-HEPES 10 and glucose 10, pH 7.4., final Na^+^ concentration 140 mmol/l). After 5 min of equilibration, cells were stimulated at 0.5, 2 and 4 Hz, and intracellular sodium levels measured for each frequency. Afterwards each cell was calibrated by adding ionophores (Gramicidin 2 µM; Monensin 40 µM; Strophantidin 100 µM) to the chamber to make the cells permeable for Na+. After a short incubation we applied modified Tyrode’s solution with different Na^+^ concentrations to acquire a calibration curve. The concentrations ranging from 0 mmol/l Na^+^ to 10, 20 and 40 mmol/l c[Na^+^] were chosen as we expected the [Na^+^]_intra_ levels within this range. Finally, the last calibration point was chosen with 145 mmol/l Na^+^, as this is in the range of normal extracellular Na^+^ levels.

[Na]_i_ results at a stimulation rate of 2 Hz were used for our final analyses, as this made the results comparable with previous publications. (Results from 0.5 and 4 Hz are presented in the Supplement).

### Ratio of total Na^+^ content to intracellular Na^+^ levels

To further characterize the relationship between [Na^+^]_i_ levels and TSC, we formed the ratio between the two parameters. Thus, when calculating [Na^+^]_i_/TSC, a higher value means a stronger dependence of TSC on intracellular sodium. Consequently, a lower value is interpreted as a stronger dependence of TSC on extracellular sodium (storage). We calculated these ratios for the mean values from all available data for [Na^+^]_i_ and TSC in the each group, as well as for the individual mice in which both values—TSC from MRI and mean [Na^+^]_i_ out of isolated CM of the respective mouse—when available.

### Calculation of extracellular Na^+^ estimates

For a more accurate estimate, we used additional data—TSC, [Na^+^]_i_ and extracellular Volume (ECV)—from the prior described experiments and we calculated an approximated total extracellular sodium content ([Na^+^]_e_) by solving the following equation:

$$TSC=ECV\times {[Na]}_{e}+{\left(1-ECV\right)\times [Na]}_{i}$$

to

$${[Na]}_{e}=\frac{TSC-{\left(1-ECV\right)\times [Na]}_{i} }{ECV}$$

with (1 − ECV) representing the intracellular volume.

Assuming that unbound extracellular sodium is in exchange with blood plasma and therefore maintains a stable level around 157 mmol/l in all mice. The difference in extracellular sodium levels between the TAC or MI model and the SHAM group is therefore a surrogate marker for the increase in bound extracellular sodium. The increase in bound extracellular sodium can be estimated with:

$$additional \,{[Na]}_{e\;bound}={[Na]}_{extra \,(Tac\;or\;MI)} -{[Na]}_{extra\;calculated\;from\;SHAM}$$

### Histological analyses

Histological Analyses Histological analyses were performed by standard techniques. Wheat-germ-agglutinin-(WGA)-DAPI staining was used for cardiomyocyte cross sectional area analyses and picrosirius red staining to examine the degree of fibrosis.

WGA-DAPI cryosections (8 µm) were examined with a Keyence microscope in fluorescent light at 20× magnification, with cell membranes fluorescing green by WGA staining and DAPI-stained nuclei fluorescing blue. Ten images per section were acquired and analyzed using Fiji/ImageJ (software version 2.1, Bethesda, USA), to cover the whole ventricle and to avoid bias by only measuring cell areas of a specific part of the heart/left ventricle. The images of both fluorescence channels were superimposed and a total of approx. 100–120 cells were selected per section and their cell area measured.

The PSR-stained paraffin-sections (7µm) were photographed using the Keyence microscope at 4× magnification in transmitted light for an overall image of the ventricle. The Fiji/ImageJ program was used to analyze the images by removing the background and determining once the total number of pixels of the left ventricle and once the number of PSR-positive pixels, i.e., above a red channel threshold set for all images. The ratio of these two numbers was calculated (number of PRS-Positive pixels/total number of pixels) and is presented as fibrosis share. For better visualization, the PSR images were converted to a false color mode.

### Statistical analyses

Results are shown as mean ± SD. One-way ANOVA followed by Tukey’s multiple comparisons test was used to compare multiple groups. To compare isolated cells nested one-way ANOVA was performed. All tests were performed using GraphPad Prism version 10 for Windows and MacOS (GraphPad, http://www.graphpad.com). P-values of < 0.05 were considered statistically significant.

Post hoc power calculations performed with G*power (Version 3.1) resulted in a power of 0.99758, based on an effect size of 1.59561 with an alpha of 0.05 and a total sample size of 14 with 3 groups.

### Supplementary Information

Supplementary Information.

## Data Availability

The datasets generated during and/or analysed during the current study are available from the corresponding author on reasonable request.
