# Supplementary material for: A new approach to characterize cardiac sodium storage by combining fluorescence photometry and magnetic resonance imaging in small animal research
Source: Sci Rep. 2024 Jan 29;14:2426. doi: 10.1038/s41598-024-52377-w (PMC10825176; doi:10.1038/s41598-024-52377-w)
Supplement: Supplementary file 1 — Supplementary Information. [file 41598_2024_52377_MOESM1_ESM.docx]

**Supplementary data**

TABLE ST1:

**Table S1:**

**(A)** Here results from the individual mice, from which complete datasets with TSC, ECV and [Na^+^]_i_ were available is shown, together with the respective calculated values.

**(B)** Not all animals in the MRI underwent cardiomyocyte isolation but were sacrificed for histology. At the bottom, the results for each group are given, derived from mean values out of all available data per group.

**(C)** Shows the MRI measurement results for sodium and ECV for the respective mice, giving an overview on the available data.

**A)**

| **mouse** | **Total sodium content (mmol/l)** | **[Na^+^]_intra_ at 2Hz stimulus (mmol/l)** | **ECV** | **Calculated [Na^+^]_extra_** | **[Na^+^]_intra_ /TSC** | **Ex =** | **In =** | **Ratio Ex/In** |  |
| --- | --- | --- | --- | --- | --- | --- | --- | --- | --- |
|  |  |  |  |  |  | **[Na^+^]_extra_ x ECV** | **[Na^+^]_intra_ x (1-ECV)** |  |  |
| SHAM1 | 41.7 | 16.7 | 0.187 | 150.8 | 0.399 | 28.2 | 13.5 | 2.08 |  |
| SHAM2 | 46.1 | 16.1 | 0.205 | 162.5 | 0.349 | 33.3 | 12.8 | 2.60 |  |
| SHAM3 | 42.5 | 17.1 | 0.234 | 125.4 | 0.404 | 29.3 | 13.1 | 2.23 |  |
| Mean SHAM | 43.4 | 16.6 | 0.209 | 146.2 | 0.384 | 30.3 | 13.2 | 2.31 |  |
|  |  |  |  |  |  |  |  |  |  |
| TAC1 | 47.1 | 23.2 | 0.178 | 157.3 | 0.495 | 28.0 | 19.2 | 1.46 |  |
| TAC2 | 50.4 | 22.0 | 0.167 | 192.7 | 0.436 | 32.1 | 18.3 | 1.75 |  |
| TAC3 | 49.8 | 21.6 | 0.250 | 134.2 | 0.435 | 33.6 | 16.2 | 2.07 |  |
| Mean TAC | 49.1 | 22.3 | 0.198 | 161.4 | 0.455 | 31.2 | 17.9 | 1.76 |  |
|  |  |  |  |  |  |  |  |  |  |
| MI1 | 67.5 | 23.8 | 0.225 | 218.4 | 0.352 | 49.0 | 18.4 | 2.66 |  |
| MI2 | 62.2 | 19.6 | 0.213 | 219.7 | 0.315 | 46.8 | 15.4 | 3.03 |  |
| MI3 | 68.6 | 25.2 | 0.183 | 262.9 | 0.367 | 48.0 | 20.6 | 2.33 |  |
| Mean MI | 66.1 | 22.9 | 0.207 | 233.7 | 0.345 | 47.9 | 18.1 | 2.67 |  |

**B)**

| Combined Data from all single measurements | | | | | | | | | |
| --- | --- | --- | --- | --- | --- | --- | --- | --- | --- |
| **mouse** | **Total sodium content (mmol/l)** | **[Na^+^]_intra_ at 2Hz stimulus (mmol/l)** | **ECV** | **Calculated [Na^+^]_extra_** | **[Na^+^]_intra_/TSC** | **Ex =** | **In =** | **Ratio Ex/In** | **[Na+]extra_bound** |
|  |  |  |  |  |  | **([Na^+^]_extra_ x ECV)** | **([Na^+^]_intra_ x (1-ECV))** |  |  |
| SHAM | 44.6 | 16.6 | 0.199 | 157.1 | 0.373 | 31.2 | 13.3 | 2.34 | 0.1 |
| TAC | 48.7 | 22.3 | 0.187 | 163.4 | 0.458 | 30.5 | 18.1 | 1.69 | 6.4 |
| MI | 63.9 | 22.9 | 0.198 | 230.3 | 0.358 | 45.6 | 18.4 | 2.48 | 73.3 |

**C)**

| **mouse** | **Total sodium content (mmol/l)** | **[Na^+^]_intra_ at 2Hz stimulus (mmol/l)** | **ECV** |
| --- | --- | --- | --- |
|  |  |  |  |
| SHAM1 | 41.7 | 16.7 | 0.187 |
| SHAM2 | 46.1 | 16.1 | 0.205 |
| SHAM3 | 42.5 | 17.1 | 0.234 |
| SHAM4 | 43.2 | n.a. | 0.207 |
| SHAM5 | 51.5 | n.a. | 0.254 |
| SHAM6 | 42.3 | n.a. | 0.106 |
| SHAM7 | n.a. | n.a. | n.a. |
| Mean SHAM | 44.567 | 16.6 | 0.199 |
|  |  |  |  |
| TAC1 | 47.1 | 23.2 | 0.178 |
| TAC2 | 50.4 | 22.0 | 0.167 |
| TAC3 | 49.8 | 21.6 | 0.250 |
| TAC4 | n.a. | n.a. | 0.143 |
| TAC5 | 47.3 | n.a. | 0.181 |
| TAC6 | n.a. | n.a. | 0.203 |
| Mean TAC | 48.7 | 22.3 | 0.187 |
|  |  |  |  |
| MI1 | 67.5 | 23.8 | 0.225 |
| MI2 | 62.2 | 19.6 | 0.213 |
| MI3 | 68.6 | 25.2 | 0.183 |
| MI4 | n.a | n.a. | 0.211 |
| MI5 | 57.4 | n.a. | 0.158 |
| Mean MI | 63.9 | 22.9 | 0.198 |

**Table ST2:**

|  | **[Na^+^]_i_** | **Hkt** | **ECV** | **ICV** | **[Na^+^]_i_ x ICV** | **TSC** | **[Na^+^]_e_** |
| --- | --- | --- | --- | --- | --- | --- | --- |
|  | 15.7 | 0.45 | 0.243 | 0.757 | 11.881 | 40 | 115.7 |
|  | 16.2 | 0.5 | 0.221 | 0.779 | 12.618 | 42.5 | 135.2 |
|  | **16.7** | **0.55** | **0.199** | **0.801** | **13.377** | **45** | 158.9 |
|  | 17.2 | 0.6 | 0.177 | 0.823 | 14.158 | 47.5 | 188.4 |
|  | 17.7 | 0.65 | 0.155 | 0.845 | 14.960 | 50 | 226.1 |
|  |  |  |  |  |  |  |  |
| **Error/ Uncertainty** | 5% | 14% | 18% | 4% | 9% | 9% | 27% |

Table ST2: Error propagation due to uncertainty on [Na^+^]_i_ and Hkt on ECV calculation. Using background noise an error of 9% over all groups on TSC quantification is calculated, this finally leads to an uncertainty of 27% für [Na^+^]_e_ defined as mean percentage variation relative to the central values. The mean [Na^+^]_i_ for SHAM mice and the selected Hkt of 0.55, with resulting values is bold.

**Figure S1**


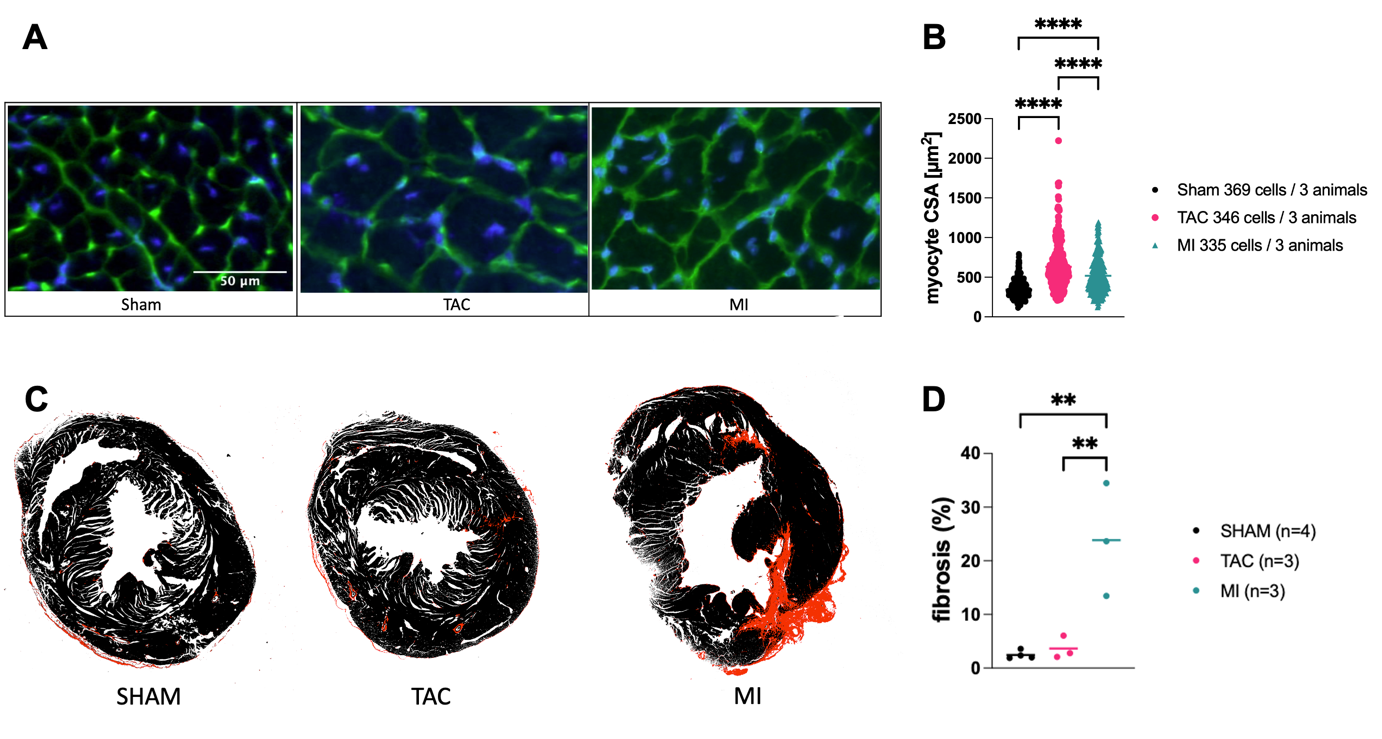


*Figure S1: Analysis of myocyte cross-sectional area and fibrosis*

*Myocyte cross-sectional area was assessed by staining myocytes with WGA and DAPI to count individual myocytes. Representative images for each treatment are shown in (A). Results of myocyte cross-sectional area analyses are presented in (B). To evaluate interstitial fibrosis in the sections were stained with PSR. Representative sections in false color mode with interstitial collagen deposits highlighted in red are depicted in (C) and the evaluation of the collagen fraction content is found in (D). * p < 0.05; ** p< 0.01; *** p< 0.001; **** p<0.0001*

**Figure S2**


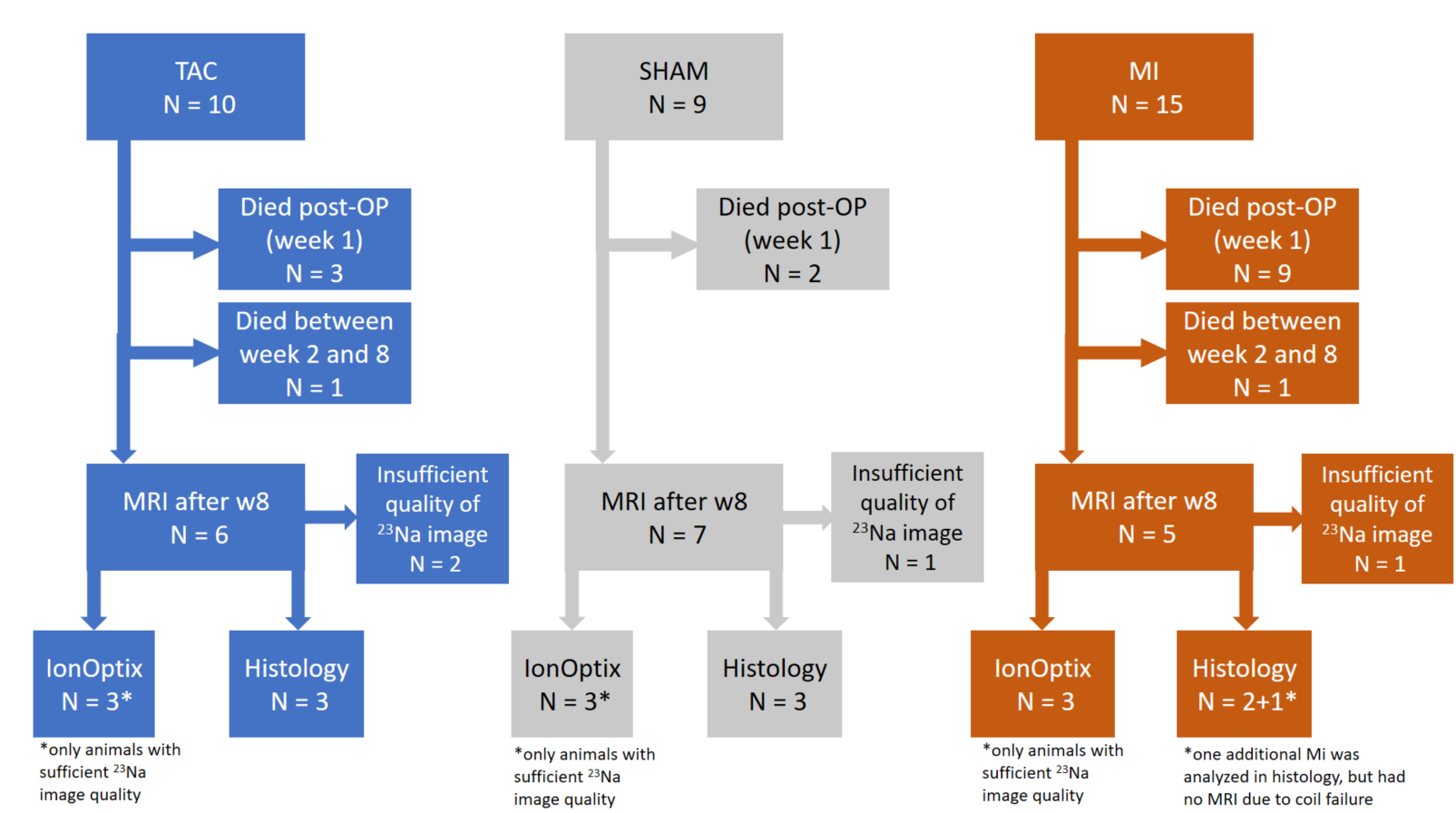


*Figure S2: Flowchart depicting the number of animals used in the study and their allocation to the respective groups and analyses.*

**Figure S3:**

**
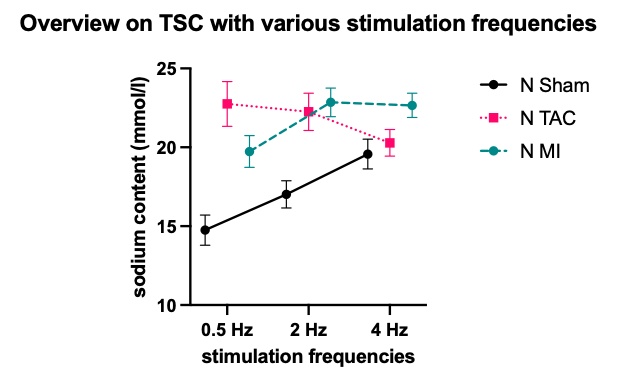
**

*Figure S3: Graphic overview of different intracellular Na^+^ levels depending on the applied stimulation frequency.*
